# Predicting general criminal recidivism in mentally disordered offenders using a random forest approach

**DOI:** 10.1186/s12888-015-0447-4

**Published:** 2015-03-29

**Authors:** Marlon O Pflueger, Irina Franke, Marc Graf, Henning Hachtel

**Affiliations:** Department of Forensic Psychiatry, University Psychiatric Clinics, Wilhelm Klein-Str. 27, CH-4012 Basel, Switzerland

**Keywords:** Criminal recidivism, Mentally disordered offenders, Risk assessment, Prediction

## Abstract

**Background:**

Psychiatric expert opinions are supposed to assess the accused individual’s risk of reoffending based on a valid scientific foundation. In contrast to specific recidivism, general recidivism has only been poorly considered in Continental Europe; we therefore aimed to develop a valid instrument for assessing the risk of general criminal recidivism of mentally ill offenders.

**Method:**

Data of 259 mentally ill offenders with a median time at risk of 107 months were analyzed and combined with the individuals’ criminal records. We derived risk factors for general criminal recidivism and classified re-offences by using a random forest approach.

**Results:**

In our sample of mentally ill offenders, 51% were reconvicted. The most important predictive factors for general criminal recidivism were: number of prior convictions, age, type of index offence, diversity of criminal history, and substance abuse. With our statistical approach we were able to correctly identify 58-95% of all reoffenders and 65-97% of all committed offences (AUC = .90).

**Conclusions:**

Our study presents a new statistical approach to forensic-psychiatric risk-assessment, allowing experts to evaluate general risk of reoffending in mentally disordered individuals, with a special focus on high-risk groups. This approach might serve not only for expert opinions in court, but also for risk management strategies and therapeutic interventions.

## Background

Psychiatric expert opinions, including risk assessments for criminal recidivism, frequently play a substantial role for the reasons of court orders – in particular if there is evidence that the accused is mentally impaired. Compared to its practical significance, the risk for general criminal recidivism in mentally disordered offenders – in contrast to the risk of delinquency in the general population of Continental Europe - is not well investigated [1]. Most of the existing studies are restricted in scope and mainly focus on the predictive validity of existing actuarial risk assessment instruments (ARAIs), or are interested in criminal recidivism with regard to a specific subgroup of offenders [1-6]. Moreover, most of the psychiatric expert opinions in German-speaking countries currently focus on the risk of specific reoffending, while rarely referring to the risk of criminal recidivism in general.

Most European legal systems stipulate expert witnesses to support the court if specific scientific expertise is required [7]. Besides assessing the offender’s mental state, the expert witness is also supposed to provide information about the risk of recidivism, i.e. the types of expected offences, degree of likeliness, and degree of dangerousness [8], the latter actually being more a legal than a psychiatric evaluation. Because of the potentially severe consequences of risk assessments, the methods being used should be standardized; instruments and analyses should be valid and reliable.

Currently, multiple ARIAs meeting those criteria were developed for the evaluation of specific recidivism risk in violent and sexual offenders [9,10]. ARAIs offer the advantage of being short and easy to implement, while having a good capability of discriminating between different populations. Such instruments can guide the examiner to identify risk factors by specifying the position of the examinee in relation to a statistically and criminologically defined group [11,12]. While established instruments for the prognosis of violent or sexual re-offences, including its scientific monitoring, are widely available [13], similar instruments concerning general recidivism in mentally disordered offenders are missing. As a consequence, this kind of recidivism might largely remain unnoticed by expert opinions within Continental Europe.

Additionally, there is evidence that post-release charges for new crimes or noncompliance with parole supervision among mentally disordered offenders are common (45 – 70%). In contrast, an essentially smaller proportion (11 – 25%) is involved in new felonies against persons and other serious crimes [14-16]. Thus, the base-rate of general reoffence in a post-release mentally disordered offender population is sufficiently high to develop reliable risk assessment procedures on sound numerical and statistical principles. We therefore analyzed data from the Basel Prognosis Cohort Study [4,17], applying a random forest as well as a random survival forest approach [18,19], which make use of both bootstrapping and jack-knife statistical techniques.

### Aims of the study

Our goal was to identify the most important predictors for general recidivism in this representative sample of mentally ill offenders, in order to provide a tool which prospectively supports valid and evidence based risk assessments in psychiatric expert opinions.

## Methods

### Participants

The study was approved by the local ethics committee of the University of Basel, Switzerland (“Ethikkommission beider Basel, EKBB”), and all participants (18+) gave written informed consent. N = 379 subjects were assessed by forensic psychiatrists and trained psychologists, all of whom were supervised by the same senior investigator. All examined individuals were subjected by court orders to forensic psychiatric evaluation, conducted by the Forensic Department of the Psychiatric Clinics of the University Basel between 1989 and 2000 [4]. Each evaluation extended on mental health, criminal responsibility, risk assessment, and the need for forensic psychiatric treatment.

Demographic (age, gender, nationality, intelligence) and forensic data were obtained using the Basel Catalogue for Risk Assessment (BCRA) [20], the Historical Clinical Risk Assessment (HCR-20) [21], and the Psychopathy Checklist-screening version (PCL-SV) [22]. The BCRA provided three outcome variables (risk for specific recidivism, risk for violent recidivism, and risk for general recidivism), where specific recidivism refers to a reoffence which matches the index offence. Additional forensic data encompassed two variables coding for the index offence (weighted and unweighted for violence), six variables coding for criminal history (diversity of criminal history, weighted and unweighted for violence, number of prior offences, number of prior convictions, prior incarceration rate, and whether the offender was actively administered by legal authorities at the time of the index offence), and two variables coding for the consequences following the conviction on the index offence charge (all kinds of institutional treatment and whether these were ongoing or completed at the end of the study or the time of the recidivism). Diagnoses were obtained by structured clinical examination according to the ICD-10 criteria and confirmed by supervision of the senior investigator. Complete criminal records of all included individuals were obtained from the Swiss Federal Register of Criminal Records at the end of the study (2006-11-27). Time at risk was calculated from the date of the index offence until the date of the first subsequently registered offence or the end of the study. Violent recidivism was defined as conviction for attempted or completed homicide, severe assault, rape, child abuse, arson, or robbery, and general recidivism as any reconviction. In total, 30 variables were coded as independent variables, while general criminal recidivism as indexed by the criminal record served as the dependent variable.

### Classification and prediction

Classification and prediction of recidivism were performed using the random forest algorithm introduced by Leo Breiman [18,19]. Since it is free of any parametric assumptions, this machine learning algorithm is appropriate in cases where the data structure might be non-linear and potentially involves complex higher-order interactions. The random forest method grows an ensemble of ‘classification and regression trees’ (CART) and combines them, initiating a majority vote to generate the classification result. Each binary decision tree is grown from a bootstrap (training) sample while leaving out one third of the cases (out-of-bag data, OOB, test sample). Moreover, it incorporates only a small number of randomly determined variables from the entire available set. In order to allow for decisions to be made, each variable is split such that the performance of the respective tree is optimized.

For classification and variable selection tasks, the random forest algorithm provides a ‘permutation accuracy importance’ measure (RFI) for each variable in the model, dedicated to rule out any variables which might not contribute to the task and to identify only the important variables. The RFI reflects changes in accuracy due to permuting a respective predictor variable. Thus, if these changes are significant, the predictor must have substantially contributed to the prediction at hand.

Recent progresses in machine learning algorithms allow the modeling of right-censored survival data within the random forest framework [20]. This progress is especially important for the analysis of events in time, such as criminal recidivism. Random survival forests provide an estimate of ensemble mortality (i.e. hazard-rate), which indexes the risk for an event to occur at a certain point in time.

For the sake of model stability we adopted an entirely data-driven model building approach which is based on two variable reduction and selection steps (maximizing RFI and minimizing the OOB prediction error by bootstrapping), and a final cross-validated ascending variable introduction strategy, in order to determine the ultimate prediction model [21,22].

### Statistics

Statistical and numerical analyses were performed by using the R environment for statistical computing version 3.0.3. Pearson’s chi-square and Fisher’s exact test were used for count data, two-sample Wilcox-test for ranked data.

The *randomForest* package version 4.6-7 and *randomForestSRC* package version 1.4 were used to conduct the variable reduction strategy and to create the ultimate survival and prediction model. The following three steps were performed so as to obtain the ultimate prediction model:

1. Determine the “random forest importance” (RFI) by 50 runs of the machine learning algorithm. Sort the variables by RFI in decreasing order and exclude the least important variables according to a CART criterion.
2. Create a nested collection of random forest models comprising the most important variables from step 1, and select those variables which are associated with the smallest out-of-bag prediction error (OOBE). Determine the OOBE by conducting 50 runs for each model (note that these first two steps were performed by using the conventional random forest approach).
3. Create a nested collection of random survival forest models comprising the most important variables from step 2, and check whether the out-of-bag prediction error (OOBE) monotonically decreases with increasingly larger models. Deploy a 4-fold cross-validation to assure valid variable selection. Determine the OOBE by conducting 20 runs for each model.

Finally, linear and non-linear regression approaches were used for post-hoc effect specifications. By means of a Receiver Operating Curve (ROC) the prediction/discrimination performance of the model was evaluated and a measure of calibration was reported.

## Results

### Sample structure

In total N = 379 individuals were examined. n = 14 were excluded from analysis because of an inconsistent sequence of events. Of the remaining n = 365 cases, n = 352 persons were convicted, n = 259 of them were detained under inpatient treatment order and/or incarcerated. Median time at risk was 107 (IQR 104) months. Due to legal reasons in most of the non-convicted (n = 13) and in those who did not receive treatment order and/or were not sentenced to arrest (n = 93), dates of index offences according to the criminal records were expunged. Consequently, these data were not considered during the analysis. Out of the remaining n = 259 offenders, n = 109 were arrested and received no treatment, n = 43 received additional treatment, n = 78 were transferred from arrest to treatment, and n = 29 were directly referred to a forensic-psychiatric setting, where treatment was mandatory.

**Figure 1:**
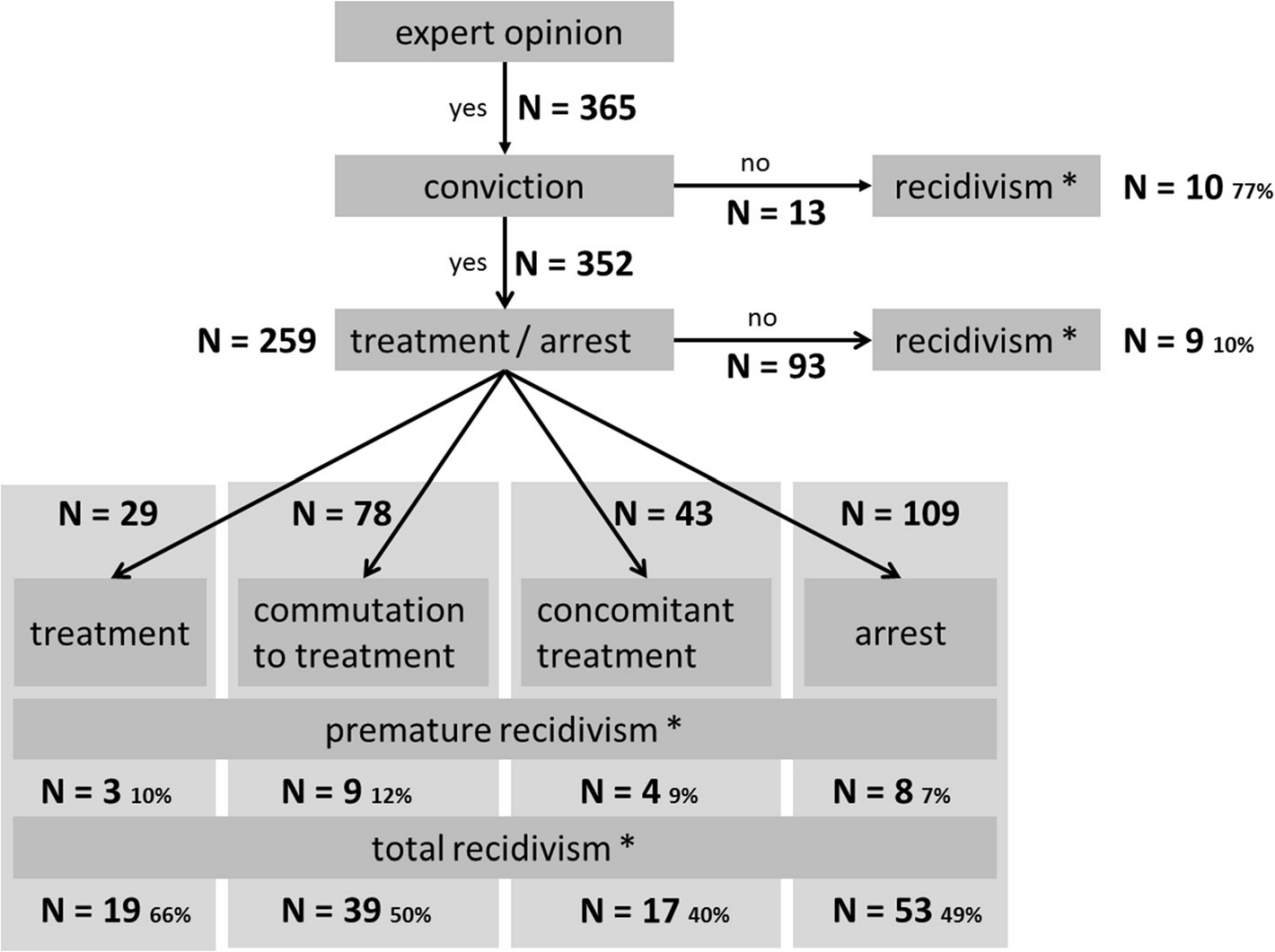
**Sample structure of the Basel Prognosis Cohort Study.** N = 365 mentally disturbed offenders were examined between 1989 and 2000. N = 352 were consecutively convicted and N = 259 were incarcerated (arrest) or received forensic therapeutic treatment (treatment). Premature recidivism refers to recidivism that occurred prior to release from jail or the end of forensic treatment.

The recidivism pattern associated with those interventions was quite stable (~51% ± 11%, cf. Figure 1). In total, from all n = 259 subjects, n = 128 showed criminal recidivism, and a subgroup of n = 17 (13%) violent recidivism.

Notably, a statistically significant (p < .001) and especially high rate of offending (77%) was observed in those n = 13 who were not convicted, as compared to the particular low recidivism rate in those n = 96 that neither received treatment nor were referred to arrest but were convicted (10%).

### Descriptive statistics

The proportion of women was 12%. There were half as many women in the recidivists-group (8%) than in the non-recidivists group (16%). Mean age at forensic-psychiatric examination was 32.6 (SD 9.9) years, with non-recidivists being roughly 6 years older than the recidivists (see Table 1). Intelligence classes were equally distributed between both groups. 40% (n = 98) of all subjects were immigrants. The proportion of recidivism in this immigrant group was approximately 55% smaller than in home country nationals. The most frequent offence-related disorders according to ICD-10 were personality disorders F6 (51%), followed by substance abuse disorders F1(49%). The prevalence of the remaining categories of disorders did not exceed 9%. A significantly higher proportion of subjects suffering from both the F1 and F6 conditions were observed within the recidivists-group (cf. Table 1). The most frequent index offences were homicide (34%), property crimes (17%), and sexual offences (16%). The incidence of other offences was below 10%. Compared to non-recidivists, recidivists were characterized by a significantly higher proportion of severe assault, property crimes, violation of narcotics law, and robbery, but were less likely involved in homicide at their index offence.

**Table 1:** **Sample characteristic of the N = 259 subjects**

	**Without recidivism**	**With recidivism**	**Statistics**
	**N = 131**	**N = 128**	
Age (yrs)*	35.5 (11.2)	29.6 (7.2)	W = 11070.5; p < .001
Gender (female %)	21 (16)	10 (8)	p = .055
Home country national (%)	66 (52)	85 (70)	p = .003
Intelligence classification (%)			
Inferior	12 (9)	3 (2)	χ^2^ = 7.7, df = 4; p = .100
Low	11 (8)	8 (6)	
Moderate	90 (69)	104 (82)	
High	11 (8)	8 (6)	
Superior	6 (5)	4 (3)	
Mental and behavioral disorders according to ICD-10			
Organic disease (%)	5 (4)	2 (2)	p = .447
Substance abuse (%)	39 (30)	87 (68)	p < .001
Schizophrenic disorders (%)	6 (5)	5 (4)	p = 1.00
Affective disorders (%)	7 (5)	3 (2)	p = .334
Somatoform disorders (%)	16 (12)	6 (5)	p = .043
Personality disorders (%)	58 (44)	74 (58)	p = .035
Mental retardation (%)	3 (2)	1 (1)	p = .622
Developmental disorder (%)	1 (1)	0 (0)	p =1.00
Disorders with onset in childhood and adolescence (%)	0 (0)	4 (3)	p = .058
Index offences			
Danger to public safety (%)	3 (2)	2 (2)	p = 1.00
Violation of narcotics law (%)	7 (5)	18 (14)	p = .021
Illegal restraints (%)	7 (5)	7 (5)	p = 1.00
Offence against life and limb other than homicide (%)	2 (2)	0 (0)	p = .498
Miscellaneous offences (%)	2 (2)	0 (0)	p = .498
Assault (%)	6 (5)	18 (14)	p = .010
Homicide (%)	67 (51)	21 (16)	p < .001
Property crimes (%)	9 (7)	35 (27)	p < .001
Robbery (%)	2 (2)	12 (9)	p = .006
Sex offence (%)	26 (20)	15 (12)	p = .089
Diversity of criminal history*	0 (1)	2 (3)	W = 5102.5; p < .001
Number of prior convictions*	0 (1)	3 (3)	W = 2411; p < .001
Number of prior offences*	0 (4)	9 (15)	W = 2636; p < .001
Duration of prior imprisonment (%)			
0 yrs	102 (78)	57 (45)	χ^2^ = 32.3, df = 2; p < .001
< 1 yrs	11 (8)	39 (30)	
>1 yrs	18 (14)	32 (25)	

*Median and interquartile range; Wilcoxon rank sum test with continuity correction.

Note: where not otherwise stated, cells contain absolute and relative frequencies; Fisher's Exact Test for Count Data.

Demographics, offence related disease, and type of offence stratified for recidivism. Behavioral syndromes associated with physiological disturbances and physical factors (ICD-10 F5) have not been observed.

The most common acts of re-offending were violation of narcotics law (26%), property crimes (22%), violation against road traffic act (19%), illegal restraints (13%), and offences against life and limb other than homicide (10%). The rate of violent recidivism acts was below 10% of all committed crimes.

50% of all subjects were considered to be at high risk of specific recidivism, i.e. they were classified as holding a high risk of repeating the index offence (cf. Table 1). In 75% of the cases, the information obtained was not sufficient to classify the subjects according to a low, average, or high risk stage for general recidivism using the BCRA. The risk for violent recidivism in recidivists was more frequently assessed than in non-recidivists. There was no difference between recidivists and non-recidivists regarding the degree of psychopathy traits, as measured with PCL-SV. However, HCR-20 indexed the recidivists as potentially bearing a higher risk of violence (cf. Table 2).

**Table 2:** **Examination results according to BCRA, psychopathic traits (PCL-SV), and violent risk assessment (HCR-20) stratified for recidivism**

	**Without recidivism**	**With recidivism**	**Statistics**
	**N = 131**	**N = 128**	
BRCA risk for specific recidivism			
Not determinable or low (%)	45 (34)	44 (34)	χ^2^ = 1.0, df = 2; p = .607
Average (%)	23 (18)	17 (13)	
High (%)	63 (48)	67 (52)	
BRCA risk for general recidivism			
Not determinable or low (%)	94 (72)	105 (82)	χ^2^ = 4.9, df = 2; p = .085
Average (%)	7 (5)	2 (2)	
High (%)	30 (23)	21 (16)	
BRCA risk for violent recidivism (%)**	104 (79)	120 (94)	p < .001
PCL total*	9 (8.5)	10 (6)	W = 7685; p = .245
HCR total*	16 (11)	18 (8)	W = 6684; p = .005

*Median and interquartile range; Wilcoxon rank sum test with continuity correction.

**According to BCRA “Risk for violent recidivism” is viewed as a dichotomous variable and does not rely on staging.

Note: where not otherwise stated, cells contain absolute and relative frequencies; Fisher's Exact Test for Count Data.

The diversity of criminal record, the number of prior offences, and, accordingly, the number/proportion of prior convictions/imprisonment in those that were involved in later recidivism was higher (median of a diversity of 2 criminal acts, 9 prior offences, 3 prior convictions, and 55% imprisonment) compared to the non-recidivists (0, 0, 0, 22%).

### Data reduction and prediction

#### Variable reduction by random forest importance maximization and OOB error minimization

During the two variable reduction and selection steps, six out of the initial 30 variables were identified as most important according to random forest RFI measure and associated with a minimal OOB prediction error. Ordered by descending RFI, these were: number of prior convictions in criminal history, prior criminal offences, age at examination, type of index offence, diversity of criminal history, and substance abuse disorder according to ICD-10 F1.

#### The prediction of general criminal recidivism with ‘random survival forest’

In order to determine a good, parsimonious prediction model, the six most important variables were sequentially entered into nested random survival forests. The OOB error was obtained by conducting a 50 fold model computation independently for each of the six nested models. Additionally, a four-fold cross validation procedure was run. Except for prior criminal offences, each variable entailed a statistically significant OOB error reduction, as indicated by cross-validation (Table 3).

**Table 3:** **Results of bootstrapped and cross-validated ascending variable introduction into a random survival forest classifier**

**Nested models**	**OOB error**	**sem**	**IOE**	**CVOE**	**CVIOE**
pcv	0.278	0.002	0.222	0.233	0.267
pcv + pco	0.276	0.002	0.003	0.255	−0.021
pcv + pco + age	0.221	0.002	0.054	0.23	0.025
pcv + pco + age + iofc	0.206	0.001	0.015	0.217	0.014
pcv + pco + age + iofc + icd1	0.204	0.002	0.002	0.211	0.005
pcv + pco + age + iofc + icd1 + div	0.197	0.002	0.007	0.193	0.019

pcv: prior convictions; pco: prior criminal offences; iofc: index offence; icd1: ICD-10 F1; div: diversity of criminal history, sem: standard error of the mean, IOE: incremental OOB error, CVOE: cross-validated OOB error, CVIOE: cross-validated incremental OOB error.

Note: the term incremental refers to the difference between two consecutive bootstrapped or cross-validated OOB classification errors.

The bootstrapped OOB classification error was strictly monotonically decreasing (cf. column 2) as additional variables were introduced and as indicated by the constantly positive incremental OOB classification error (cf. column 4). An OOB classification error of 0.5 corresponds to tossing a coin. Except for prior criminal offences, each additional variable was also associated with a cross-validated OOB error reduction (cf. columns 5, 6).

**Figure 2:**
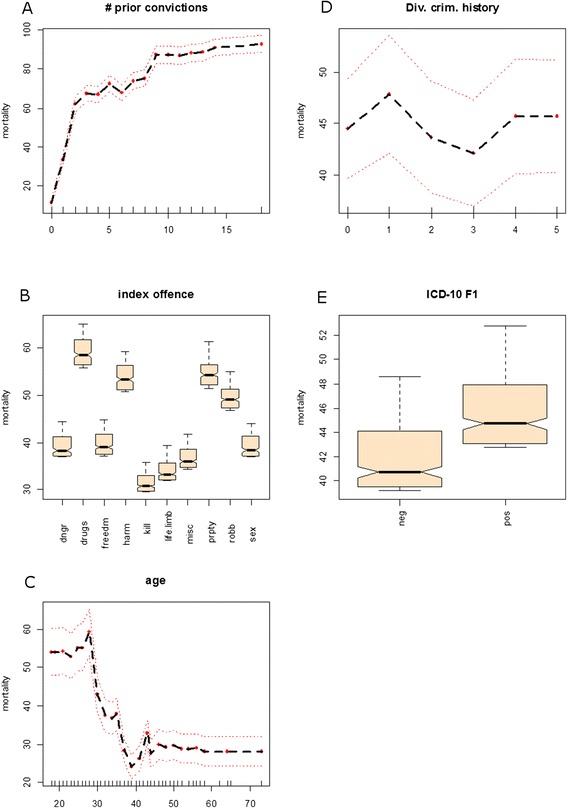
**Average partial OOB ensemble mortality (OOBEM, i.e. a hazard-rate) as function of A) prior convictions, B) kind of index offences committed, C) age at index offence, D) diversity of criminal history, and E) substance abuse (according to WHO ICD-10).** The variables are ordered by importance. The average partial ensemble mortality is predicted by the Random Survival Forest. All effects are adjusted for one another. dngr: danger to public safety, drugs: violation of narcotic laws, freedm: illegal restraints, life.limb: Offence against life and limb other than homicide, misc: miscellaneous offences, harm: assault, kill: homicide, prpty: property crimes, robb: robbery, sex: sex offence.

The number of prior criminal offences was dropped from the model since it was highly correlated with the number of prior convictions as indicated by the cross-validation procedure. The resultant five most important predictors contributed differentially and in a non-linear way to the average partial OOB ensemble mortality (OOBEM, i.e. hazard-rate) (see Figure 2). The number of prior convictions contributed most to OOBEM. In fact, OOBEM increased as a square root function of the number of prior convictions by a slope of 19.4% and an intercept of 23.6% (F1,14 = 135.6, p < .001). Basically, offenders committing rather less serious index offences, such as violation of narcotics law (59%), property crimes (54%), assault (53%), and robbery (49%), were tightly associated with an elevated risk for reoffending (remaining index offences entailed an OOBEM between 30% and 40%). Other predictors such as age below 30 years (<30ys OOBEM ~ 54%, > 30ys OOBEM ~ 30%; F2,22 = 150.5, p < .001), substance abuse (increased OOBEM by 4%), and the diversity of criminal history (cyclic deflection of ±2.9% from baseline at 44.2% OOBEM; F2,3 = 9.1; p = .053) contributed, likewise, significantly. According to time at risk, a 25%, 50%, 75%, and 100% recidivism (hazard) rate was observed after approximately 7, 10, 12, and 14.5 year’s observation period.

As with the former identified predictors, 13 variables derived from structured risk assessments (HCR-20, PCL-SV and BCRA) and ordered by RFI were likewise entered into sequentially nested random survival forests. Unlike the five most important predictors, the nested risk assessment models could not reduce the OOB classification error substantially below 0.4. In terms of prediction accuracy, an OOB classification error of 0.5 corresponds to tossing a coin.

Concerning the final model, the OOBEM was extracted and a Receiver Operating Characteristic (ROC) was constructed. Three cut-off values were derived according to different weightings of sensitivity and specificity. The first considered sensitivity and specificity to be equally weighted. In the second and third case, either sensitivity (sens.) or specificity (spec.) was weighted as to encompass 95% of the respective recidivists/non-recidivists.

The area under the curve (AUC) summed up to .90 which is sufficiently acceptable in terms of prediction performance. The first cut-off value (33.1) resulted in 85% overall accuracy-rate (sens. 0.84, spec. 0.86) and accounted for 91% of the observed reoffences. N = 15 out of N = 17 violent recidivists were correctly classified. The 95% sensitivity cut-off (7.3) yielded a 77% overall accuracy-rate (spec. 0.60) and accounted for 97% observed reoffences. All N = 17 violent recidivists were identified. And, finally, the 95% specificity cut-off (69.7) entailed a 77% overall accuracy-rate (sens. 0.58) and accounted for 65% of all reoffences. This time, N = 12 out of 17 violent reoffenders were correctly indexed. Thus, even if opting for the most conservative predictive approach, the model nevertheless succeeds in explaining a substantial proportion of general and violent reoffences.

Regardless of a particular cut-off value, the predicted reoffence characteristic was dominated by offences against life and limb other than homicide, illegal restraints, violation against road traffic act, property crimes, and violation of narcotics law. Severe reoffences were less frequently predicted, which was in accordance to the reoffence characteristics observed in general population.

Table 4 shows probabilities of recidivism related to risk categories as derived from the aforementioned cut-off values. The probabilities serve as calibration measure for the ultimate random survival forest prediction model. We refer to four categories of increasing risk classes, ranging from the lowest p < 10% to the highest p > 90% recidivism probability, and two intermediate risk classes. Even though the probabilities of recidivism lack equidistance, we still feel that the calibration of the risk categories is not unduly biased.

**Table 4:** **Probability of recidivism according to four risk categories derived by the random survival forest prediction model and three different cut-off values**

**Risk**	**Without recidivism**		**With recidivism**		**p (recidivism)**	
	**(N = 131)**		**(N = 128)**			
Low (%)	76	(58)	6	(5)	0.073	95% specificity
Moderate (%)	31	(24)	11	(9)	0.262	50% specificity & 50% sensitivity
High (%)	18	(14)	35	(27)	0.660	
Very high (%)	6	(05)	76	(59)	0.927	95% sensitivity

## Discussion

Previous research showed that general criminal recidivism is common in mentally disordered offenders [14-16]. We reported here that this holds as well for the Basel cohort examined between 1989 and 2000.

To this end we opted for a new and innovative approach in developing a prediction model for general recidivism using the random forest and random survival forest algorithm which are both based on a collection of decision/classification trees and especially suited for non-linear and complex data structures. Bootstrapping and jack-knife methods optimize predictive validity rather than goodness-of-fit. Since random forest procedures make ample use of both techniques, we claim that our model might have the potential to assist expert witness services in judging the risk of general criminal recidivism.

The most important variables in predicting general criminal recidivism in our sample were number of prior convictions, age at examination, type of index offence, diversity of criminal history, and substance abuse. Apart from substance abuse, which is not exclusively seen in mentally ill recidivists [6,14], no further clinical variables significantly contributed to predicting recidivism.

Our data is largely in line with previous studies mainly from the US and Canada [14-16]. As with the sample structure (general vs. violent recidivism rate), the predictor pattern largely overlapped and no predictor especially reflected clinical psychiatric significance. However, this does not mean that forensic-correctional settings have no impact on preventing recidivism, since the base-rate of violent recidivism was low and general recidivism was out of scope.

Our numerical approach yielded an acceptable performance in terms of prediction accuracy. Dependent on the particular realization of an ensemble mortality cut-off value, the presented model is able to capture between 58% and 95% of all reoffenders and between 65% and 97% of all reoffences committed. According to the four risk classes derived from the three abovementioned cut-off values, the probabilities of recidivism lack equidistance, and, yet, we feel that the risk categories are sufficiently well calibrated.

However, some caveats should be noted. Two specific subgroups of mental disorders are very prominent in our sample: personality disorders and substance abuse disorders. Due to the small number of subjects suffering from schizophrenia (11%), our results are not applicable to offenders suffering from psychotic disorders.

The biased ratio of foreign nationals versus home country nationals in the recidivism group may result from migration and expulsion of foreign nationals. Similarly, no data were obtained controlling for hospitalization or death incidents during incarceration or forensic treatment. The proportion of mentally disordered female offenders was approximately 12%, which is basically in concordance with evidence from the Swiss federal Statistic Office about the proportion of women’s convictions in the general population. Yet the female subsample is too small to have an appropriate impact on the variable selection procedure preceding the final modeling step. The same is true with violent recidivism: even though the prediction and, hence, the prevention of violent reoffences in particular would be appreciated, the base rate of its occurrence does not allow for establishing a prediction model with sufficient robustness and stability as necessary to derive reliable prognostic results. However, because felonies typically result in long-term imprisonment, where further recidivism during the observation period is less likely, it is not clear whether, or to what extent, the base-rate of violent reoffences might increase as a function of time at risk. A weak relationship would definitely impose stronger constraints on the duration of observation periods and, hence, modeling of violent reoffences might become an issue of long-term observational studies.

Despite a sufficiently good performance of our prediction model, age at examination, substance abuse disorder, type of offence, diversity of criminal history, and prior convictions are most probably not causative of criminal recidivism per se. As with all complex behaviors, criminal acts are a consequence of genetic, biological, social, and environmental factors. Therefore, the additional consideration of factors such as type and efficacy of and engagement in therapeutic intervention, the achievement of developmental milestones, socioeconomic state, as well as environmental contexts, might have reinforced prediction accuracy even more. Unfortunately, the Basel Cohort Study did not provide these data.

The high-risk recidivism group comprised rather young subjects, with conspicuous criminal history, suffering from substance abuse disorder. Moreover, the pattern of index offences in the recidivists revealed less serious criminal acts than those seen in non-recidivists. And even though the overall pattern of index offences was characterized by felonies, serious violent crimes were rarely observed later on.

The practical application of the prediction model depends on a concrete choice of an ensemble mortality cut-off value. However, this should be considered with caution. Since the numerical model generates far from perfect predictions, a potential user should bear in mind that there is a trade-off between sensitivity and specificity. Because of legal and ethical reasons, we recommend a conservative approach in determining a cut-off value by optimizing specificity. In doing so, we are focusing on a maximal risk group which encompasses 71% of the observed violent recidivists. Since the model’s usability is limited due to the lack of gender as risk factor [15,16,23], we can only assess male examinees. But apart from that, by knowing a particular examinee’s index offence, his age, substance abuse disorder, and criminal history, we can now predict whether he is likely to reoffend by determining the predicted ensemble mortality and examining whether it increases beyond a threshold of 69.7.

In defining a high risk group of mentally disordered offenders we might focus on a group of offenders who are in need of a higher degree of care and medical support in order to prevent adverse development trajectories (e.g. worsening clinical outcome, increasing personality deficits, and cognitive decline), which in turn might, in the long run, end in violent crimes. Thus, we also achieve an additional, and even more important goal, in minimizing future victimization and associated secondary costs [24].

However, to achieve this goal, prognosis research in forensic psychiatry needs to replicate these studies with a more refined data acquisition, in terms of a more thorough consideration of therapy outcomes and explicit environmental and socioeconomical data. Moreover, larger samples might find gender more appropriately considered by a numerical approach, like we did. And, finally, longer observation periods are desired as to better deal with the low violent recidivism base-rate [25].

## Conclusion

It is important to note, that a numerical and statistical prediction model cannot substitute the way legal prognoses are obtained. However, this type of model can serve as an improvement in providing integrated and summarized information, which might facilitate decision processes required for expert witness services and jurisdiction.
